# Plant Phenotyping Research Trends, a Science Mapping Approach

**DOI:** 10.3389/fpls.2018.01933

**Published:** 2019-01-07

**Authors:** Corrado Costa, Ulrich Schurr, Francesco Loreto, Paolo Menesatti, Sebastien Carpentier

**Affiliations:** ^1^Consiglio per la Ricerca in Agricoltura e l'analisi dell'economia Agraria–Centro di Ricerca Ingegneria e Trasformazioni Agroalimentari, Rome, Italy; ^2^Forschungszentrum Jülich, IBG-2: Plant Sciences, Jülich, Germany; ^3^Dipartimento di Scienze Bio-Agroalimentari, Consiglio Nazionale Delle Ricerche, Rome, Italy; ^4^Bioversity International, Genetic Resources, Leuven, Belgium; ^5^KU Leuven, Leuven, Belgium

**Keywords:** phenotyping, bibliometric analyses, imaging, growth, marker

## Abstract

Modern plant phenotyping, often using non-invasive technologies and digital technologies, is an emerging science and provides essential information on how genetics, epigenetics, environmental pressures, and crop management (farming) can guide selection toward productive plants suitable for their environment. Thus, phenotyping is at the forefront of future plant breeding. Bibliometric science mapping is a quantitative method that analyzes scientific publications throughout the terms present in their title, abstract, and keywords. The aim of this mapping exercise is to observe trends and identify research opportunities. This allows us to analyze the evolution of phenotyping research and to predict emerging topics of this discipline. A total of 1,827 scientific publications fitted our search method over the last 20 years. During the period 1997–2006, the total number of publications was only around 6.1%. The number of publications increased more steeply after 2010, boosted by the overcoming of technological bias and by a set of key developments at hard and software level (image analysis and data storage management, automation and robotics). Cluster analysis evidenced three main groups linked to genetics, physiology, and imaging. Mainly the model plant “*Arabidopsis thaliana*” and the crops “rice” and “triticum” species were investigated in the literature. The last two species were studied when addressing “plant breeding,” and “genomic selection.” However, currently the trend goes toward a higher diversity of phenotyped crops and research in the field. The application of plant phenotyping in the field is still under rapid development and this application has strong linkages with precision agriculture. EU co-authors were involved in 41.8% of the analyzed papers, followed by USA (15.4%), Australia (6.0%), and India (5.6%). Within the EU, coauthors were mainly affiliated in Germany (35.8%), France (23.7%), and United Kingdom (18.4%). Time seems right for new opportunities to incentivize research on more crops, in real field conditions, and to spread knowledge toward more countries, including emerging economies. Science mapping offers the possibility to get insights into a wide amount of bibliographic information, making them more manageable, attractive, and easy to serve science policy makers, stakeholders, and research managers.

## Introduction

Phenotyping is the foundation of any breeding selection process. However, modern plant phenotyping measures complex traits related to growth, yield, and adaptation to stress, with an improved accuracy and precision at different scales of organization, from organs to canopies (Fiorani and Schurr, 2013). A more recent and comprehensive definition for plant phenotyping is the assessment of complex plant traits such as growth, development, tolerance, resistance, architecture, physiology, ecology, yield, and the basic measurement of individual quantitative parameters that form the basis for complex trait assessment (Li et al., 2014). The plant phenotype emerges from the dynamic and local interaction of phenotypes with the spatially and temporally dynamic environment above and below ground. Direct quantification of the phenotype includes diverse structural and functional aspects, like plant biomass (Menzel et al., 2009; Golzarian et al., 2011), root morphology (Walter et al., 2009, 2015; Clark et al., 2011; Flavel et al., 2012; Kumar et al., 2014), leaf characteristics (Jansen et al., 2009; Arvidsson et al., 2011), fruit traits (Costa et al., 2011; Antonucci et al., 2012; Monforte et al., 2014), but also chemical phenotypes, like secondary metabolites with roles in plant defense and biosphere-atmosphere interactions, such as Volatile Organic Compounds (VOCs) (Taiti et al., 2015). Even more complex are functional trait concepts, like photosynthetic efficiency (Bauriegel et al., 2011), and biotic and abiotic stress resistance/tolerance (Antonucci et al., 2013; Rao and Laxman, 2013).

A major challenge in integrating such a diversity of disciplines (spanning biological sciences, computer science, mathematics and engineering) is to find a common language basis to ease communication between technology developers, providers of infrastructures and diverse users. In this *scenario* that sees phenotyping at the forefront of future plant breeding and selection, application of bibliometric science mapping is a powerful tool to observe trends, and identify research opportunities. Research trends are being detected by bibliometrics and by quantitative methods that analyze scientific publications as an information process. Once patterns and dynamics in scientific publications are identified, they are used as a proxy for the development of the investigated disciplines (Pritchard, 1969; van Raan, 2004). Thus, bibliographic analysis is a powerful tool to measure scientific production of research and trends, facilitating science-based strategy developments and policies. The bibliometric approach has successfully been applied to research fields such as climate change (Li et al., 2011), geo-statistics (Hengl et al., 2009), and solid waste research (Mesdaghinia et al., 2015). In addition, during the last years bibliometrics has been used in concert with the analysis of terms (i.e., words) that appear in the titles, abstracts and keywords of published papers. Such an approach, termed “bibliometric mapping,” has been widely used to acquire a deeper understanding on the structure of the research itself. For example, it has been applied successfully to different research fields such as the evolution of chemistry research (Boyack et al., 2008), Mediterranean forest inventories (Nardi et al., 2016), and the use of stable carbon isotopes to detect the physiological impact of forest management (Di Matteo et al., 2016). To our knowledge, bibliometric mapping or science mapping has never been applied to analyze developments in plant phenotyping. As plant phenotyping is such an emerging research subject, the aim of this analysis was to show the evolution of the plant phenotyping research structure and predict emerging topics of this discipline. This will help orienting technology providers, researchers, and end-users entering the field and will provide guidance to research managers and policy decision makers.

## Materials and Methods

### Database Search

The Scopus database was consulted on April 5th 2018 and used to retrieve bibliographic records related to plant phenotyping research for the period 1997–2017. To identify relevant plant phenotyping publications, the following keywords were used in the combined fields of title, abstract, and keywords (*per* publication): plant PRE/3 phenotyping (PRE/n means “precedes by,” where the first term in the query must precede the second by a specified number of terms n). Being the Scopus search conducted on April 2018, 2018 publications were not included in the analysis, and 2017 publications were not yet completely introduced in the Scopus database by the Scopus staff and may be underestimated. The search was restricted to publications (Article, Review, Book Chapter, Book, Letter, and Note) written in English. The EndNote file is attached as Supplementary Material.

### Bibliometric Mapping and Clustering

A general quantitative description of the bibliographic records was conducted. The trend in plant phenotyping publications from 1997 to 2017 was analyzed and compared with trends in other subjects: Genomics, Proteomics, Mediterranean forest research (Nardi et al., 2016), and Precision agriculture (Pallottino et al., 2018). Maps of the plant phenotyping publications in the world and in EU countries were constructed using color intensities related to the number of publications. Data were normalized by dividing the plant phenotyping publications *per* population number (multiplied *per* 10^7^) in the world and in EU countries (ONU, 2017). The number of publications was counted considering all the co-authors of a paper.

Bibliometric maps were created on retrieved publications, using the VOSviewer software version 1.6.5.0 (freely available at www.vosviewer.com). The software was specifically developed for creating, visualizing, and exploring science's bibliometric maps (van Eck and Waltman, 2010). Using VOSviewer we produced *term map*s. A *term map*, also called co-word map, is a two-dimensional representation of a research field, in which strongly related terms are located close to each other and the weaker the relationship is between terms, the bigger the distance is between them. Thus, term maps provide overviews for identifying the structure of a topic. Thanks to natural language processing techniques and a linguistic filter employed by the software, terms occurring in titles, abstracts and keywords of publications were extracted, and represented in the map as circles (van Eck and Waltman, 2011). Only terms occurring at least 10 times were extracted from the retrieved publications. To display the elements on maps, the software uses the VOS (*Visualization Of Similarities*) mapping technique, that is closely related to the multidimensional scaling method (van Eck and Waltman, 2010). The idea of the VOS mapping technique is to minimize a weighted sum of squared Euclidean distances between all pairs of items through an optimization process. This mapping approach allows aligning terms on the map in a way that the distance between each pair of terms represents their similarity as accurately as possible. In a *term map*, similarities among terms are calculated based on their number of co-occurrences in the title or abstract of the same publication [for further explanation on the method see van Eck and Waltman, 2010; Nardi et al., 2016]. The larger the number of publications, in which two terms co-occur, the stronger the terms are related to each other. Therefore, terms that often co-occur in the same publications are located close to each other in a term map and less strongly related terms (low co-occurrence) are located further away from each other. Each term is represented by a circle, where its diameter and the size of its label indicate the number of publications, where the term appears in title, abstract, or keywords. Once terms are in the map, the next step is to identify clusters of related terms. The software uses a weighted and parameterized variant of modularity-based clustering called VOS clustering technique (Waltman et al., 2010; Waltman and van Eck, 2013). The assignment of terms to the same cluster depends on their co-occurrences in the title or abstract of publications. More specifically, terms that often co-occur are strongly related to each other and are assigned automatically to the same cluster. On the contrary, terms with a low co-occurrence or no-occurrences at all, are assigned to different clusters. A cluster that is made up of terms of the same colors represents a research theme in which one or more research topics can be identified. Although VOSviewer offers the possibility to change the number of clusters by changing the resolution parameters, we used the default setting of one. The same approach has been applied on the bibliographic information on the same publication dataset in order to observe the countries of the co-authorship map with the aim of observing the collaborative clustering of the countries based on plant phenotyping publications.

In addition to the cluster maps, one of which was based on the entire dataset and the top 5 EU countries (Germany, France, United Kingdom, Italy, Spain), we also produced a *term citation* and *a term year map*. A term citation map analyzes the scientific impact of specific topic, whereas a term year map performs a timeline analysis of the research topics. More specifically, in the *term citation map*, the color of a term is determined by the average citation impact of the publications where the term occurs, thus reflecting the average citation impact for the term rather than by cluster (as in the *term map*). To avoid biases related to the age of a publication (older publications are expected to be more cited), the number of citations of each publication is divided by the average number of citations of all publications appearing in the same year. This produces a publication's normalized citation score range from 0 to 2. In the map, the colors are assigned according to the score, ranging from blue (average score of 0) to green (average score of 1) to red (average score of 2). Therefore, a blue (cold) or red (hot) term indicates that publications, in which the term have low and high average citation impacts, respectively (van Eck et al., 2013). In the case of *term year maps*, the color of a term indicates the average publication year of all the publications, in which the term occurs. As for the *term citation map*, we used colors that range from blue (mean year term presence 2011 or earlier), to green (2013–2014) to red (2016 or later). Therefore, blue terms are those occurring mainly in older publications, while red terms occur mainly in publications that are more recent. To avoid overlapping labels, only a subset of all labels is displayed in the maps. In order to navigate all the map with labels VosViewer Map and Network files are available as Supplementary Material.

A collaborative clustering map of the countries based on plant phenotyping publications was also produced reporting the spatial relationship among countries. Moreover, a term year map based on countries' collaboration on plant phenotyping researches was produced. Based on FAO statistics for each country, the number of research papers on plant phenotyping was compared with the percentage of total cultivated area with respect to the total area.

Before starting with the analysis in VOSviewer, a thesaurus file (text file attached as Supplementary Material) was created to ensure consistency for different term spelling and synonyms (an example: quantitative trait loci analysis is often termed qtl). VOSviewer also offers the possibility to clean the data by omitting those terms considered not relevant for analyses. Using this software functionality, we performed the cleaning by omitting terms related to time, publishers' names, and geographical locations (i.e., names of cities or countries) or terms that could be used ambiguously (an example: addition or view). It should be noticed, however, that a *term map* represents a simplified version of reality and this can lead to loss of information and to a partial representation of the investigated field (van Raan, 2014). This limitation should be considered when interpreting results.

## Results and Discussion

### Plant Phenotyping Publication Trends

**Figure 1:**
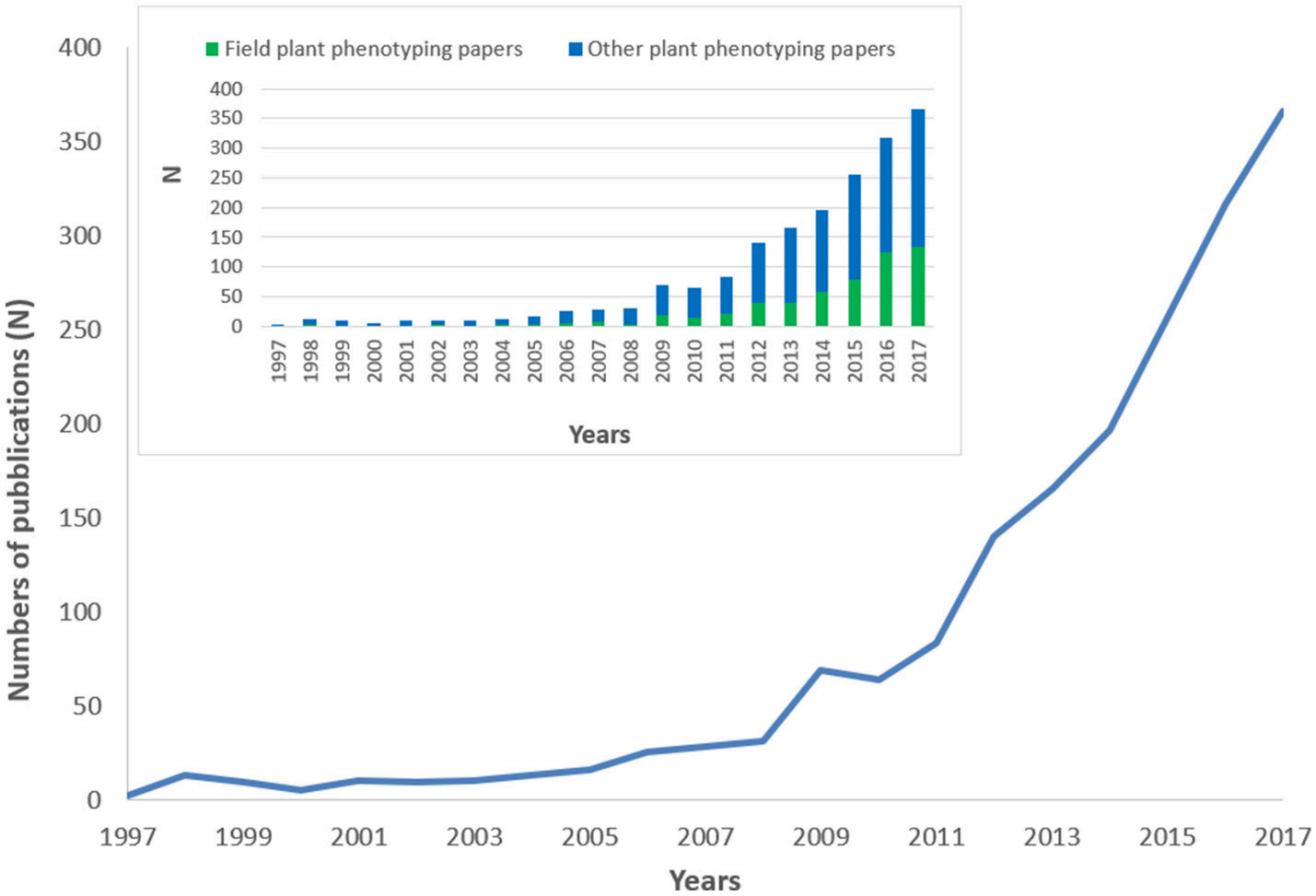
Trend in plant phenotyping publications from 1997 to 2017. The histogram on the top-left side of the figure represents the number of field plant phenotyping papers with respect to other plant phenotyping papers. 2017 publications were underestimated being not yet all inserted in the Scopus database.

A total of 1,827 scientific publications fitted our search method based on relevance for plant phenotyping. 81.3% of the publications were research papers, 11.7% review papers, 5.9% book chapters and the remaining 1.1% were books, letters, or notes. The studies were published in 145 journals. Top journals were Frontiers in Plant Science (*N* = 87; 4.8%), Journal of Experimental Botany (*N* = 83; 4.5%), Plant Methods (*N* = 72; 3.9%), Theoretical and Applied Genetics (*N* = 70; 3.8%), and *PLoS ONE* (*N* = 61; 3.3%). Figure 1 depicts the plant phenotyping publication numbers from 1997 to 2017. During the period 1997–2006, the total number of publications was only around 6.1%. The number of publications generally increased more steeply after 2010, but a first surge was already observed during 2008–2009, in coincidence with the 1st International plant phenotyping Conference 2008 in Canberra, Australia. In Figure 1 the number of field plant phenotyping papers with respect to other plant phenotyping papers was also reported. It is striking to observe how field plant phenotyping papers increases their importance since 2014 from a very small percentage in the early years to >30% of the total publications in 2016/2017.

**Figure 2:**
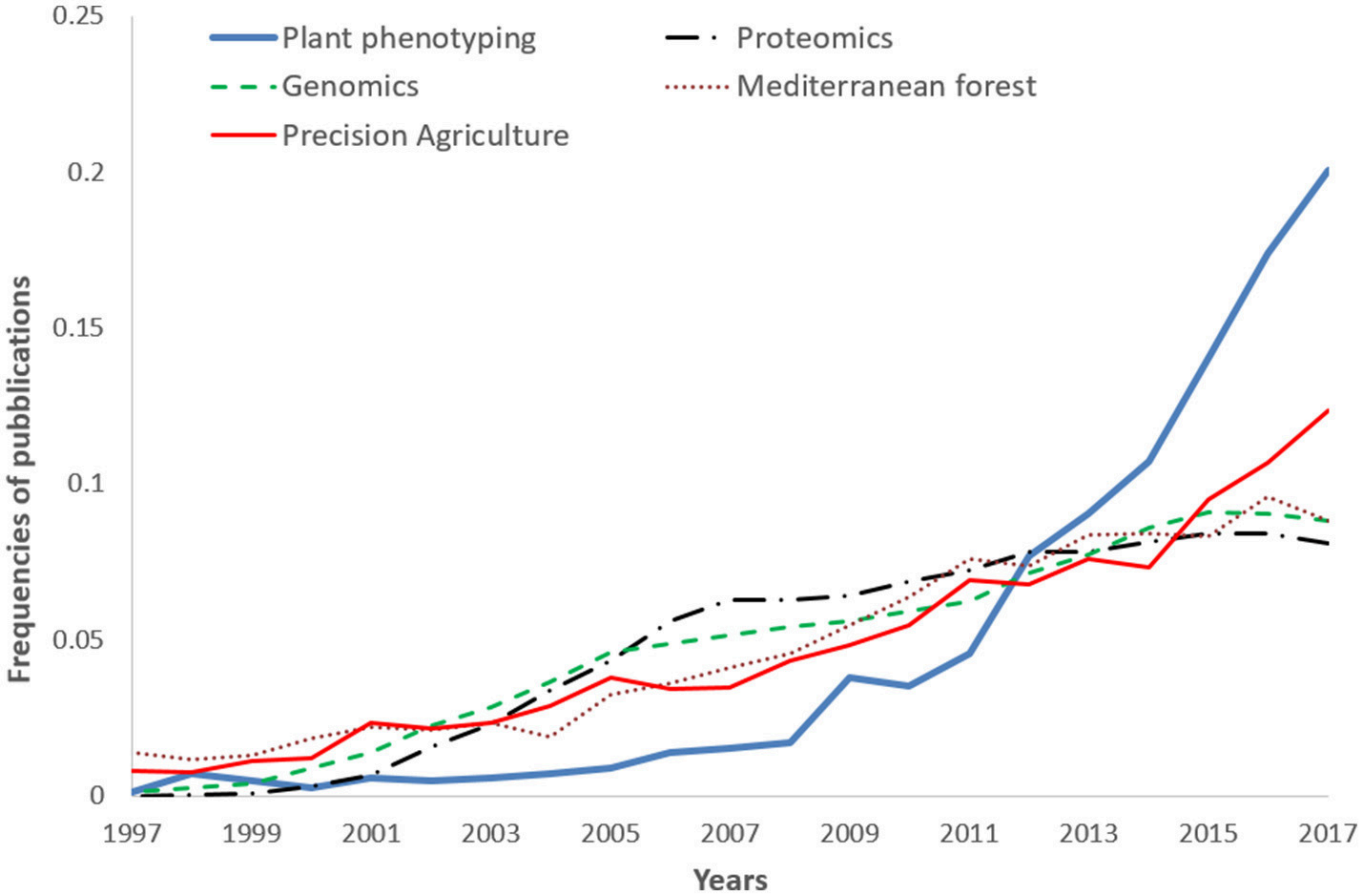
Frequencies of plant phenotyping publications from 1997 to 2017 compared with other subjects: Genomics, Proteomics, Mediterranean forest (Nardi et al., 2016), and Precision agriculture (Pallottino et al., 2018). 2017 publications were underestimated being not yet all inserted in the Scopus database.

A comparison (on standardized data) of the trend reported in Figure 1, with other disciplines like –omics (proteomics genomics), precision agriculture, and Mediterranean forestry shows the over-proportional increase of plant phenotyping papers after 2009 (Figure 2). Proteomics and genomics show a saturation trend after 2005–2006 in terms of published papers, while precision agriculture, sometimes considered a “sister discipline” of plant phenotyping, also showed a less evident increase after 2007. The application of plant phenotyping in the field is still under rapid development and this application has strong linkages with precision agriculture.

**Figure 3:**
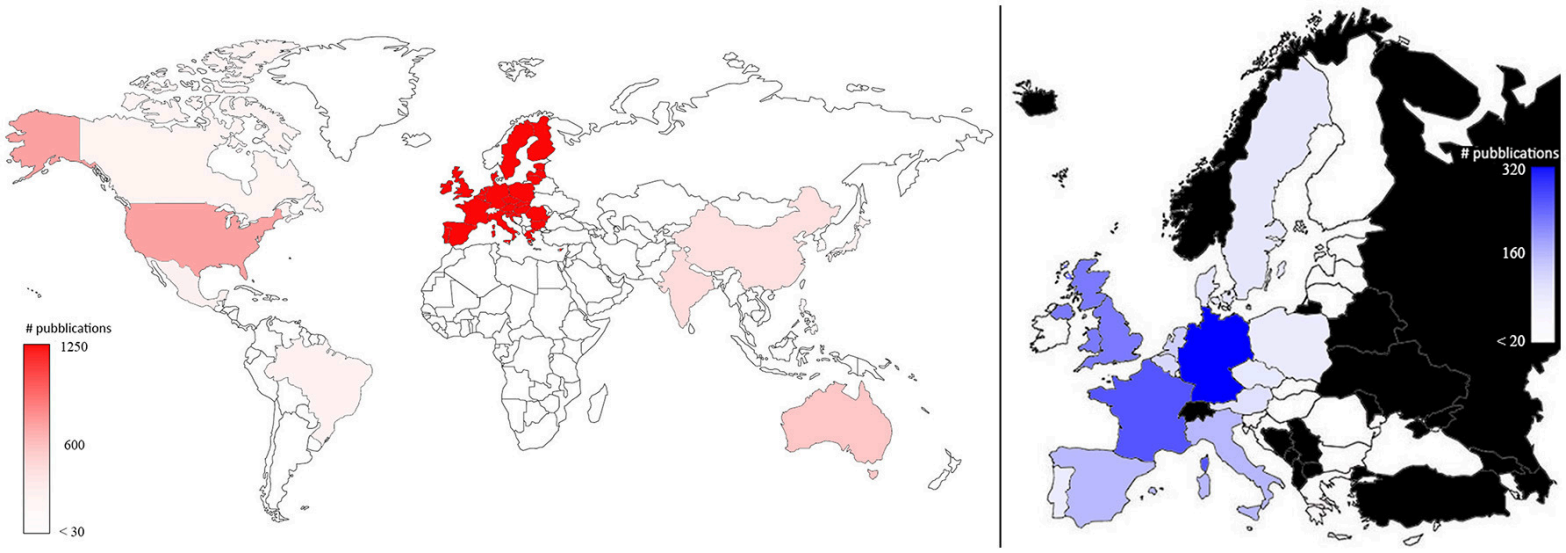
Map of the plant phenotyping publications by all the co-authors in the world (**left**) and in the EU countries (**right**; in black countries that are not part of EU). The color intensities were related to the number of publications, as highlighted in the bars.

Plant phenotyping research was published by authors working in 74 different countries. Figure 3 represents the number of papers dealing with plant phenotyping published by co-authors in the world (left) and in the EU (right). EU co-authors published 1,210 papers (41.8% of total publications considering all the co-authors) followed by USA (15.4%; *N* = 447), Australia (6.0%; *N* = 174), and India (5.6%; *N* = 161). More than 40% of the studies performed in the USA and Australia apply plant phenotyping in the field. In the EU, Germany published 35.8% (319) of the total publications, followed by France (23.7%; *N* = 211), United Kingdom (18.4%, *N* = 164), Italy (9.9%, *N* = 88), and Spain (9.5%, *N* = 85). The proportion of phenotyping dedicated to field phenotyping in those countries ranged from 31% (France) to 38% (Spain). (Pallottino et al., 2018) showed how the leading countries in precision agriculture are EU, USA China and Australia. This is comparable to what was observed in this paper for plant phenotyping (Figure 3).

**Figure 4:**
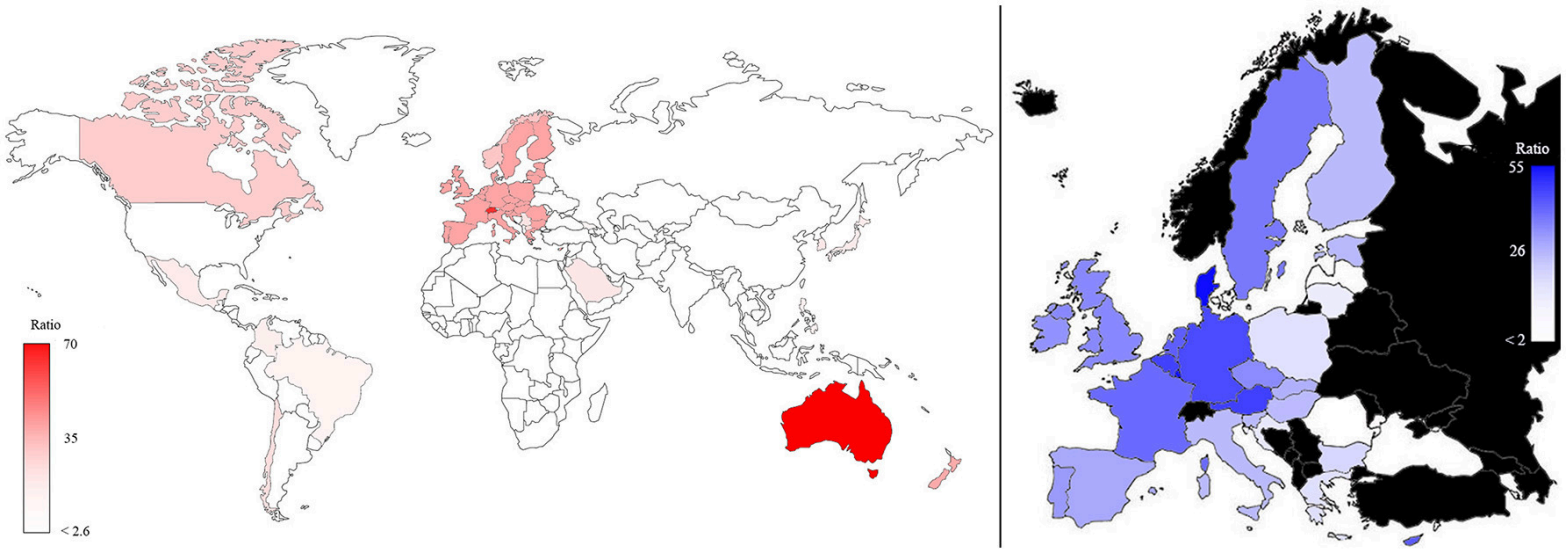
Map of the plant phenotyping publications normalized according to the number of inhabitants in the world (**left**) and in the EU countries (**right**; in black countries that are not part of EU). The color intensities were related to the number of normalized publications, as highlighted in the bars.

The same map, when standardized by dividing the plant phenotyping publications *per* population and multiplying the number *per* 10^7^ (Figure 4), shows that Australia and Switzerland are the top world countries (left) most active in phenotyping publishing relative to their population size, EU remains at a leading position (5th) while India and USA only hold the 42nd and 68th position, respectively. In EU (right side of Figure 4) Denmark and Luxembourg are the top countries, and Germany also has a leading position (5th).

### World Evolution of Plant Phenotyping Research Topics and Their Citation Impacts

**Figure 5:**
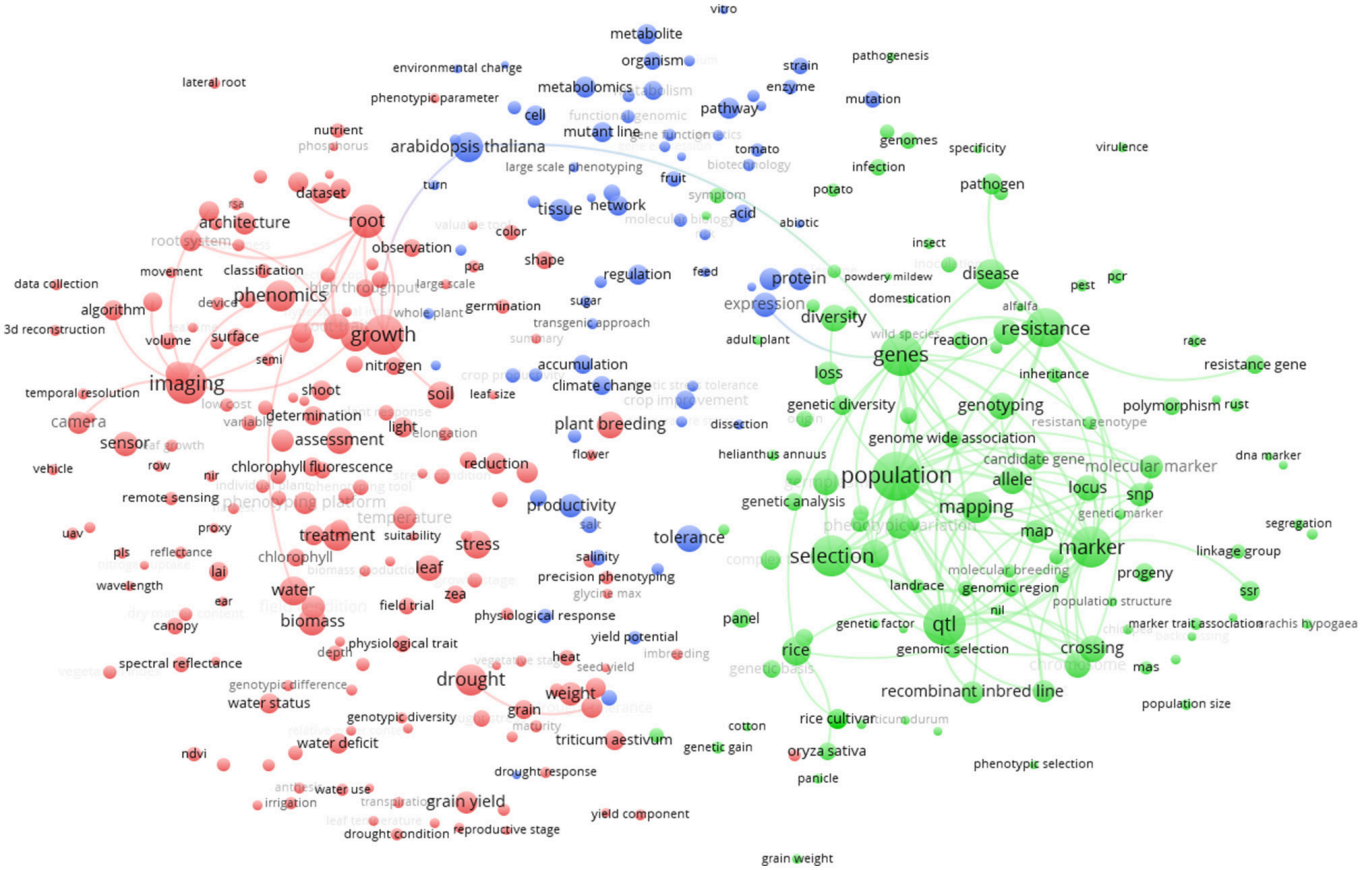
Term clustering map based on the Scopus plant phenotyping publications. Different colors (red, green, blue) represent the terms belonging to different clusters. The size of the term is based on the number of occurrences. The connecting lines indicate the 100 strongest co-occurrence links between terms.

In the world *term map* (Figure 5) the 357 terms displayed on the map are grouped in three clusters. The red cluster (174 terms) at the left side of the figure is mainly represented by the terms “imaging,” “growth,” and “phenomics.” Other important terms that cluster in red are “drought,” “root,” and “biomass.” The green cluster (113 terms) at the right-side of Figure 5 shows as main terms “selection” and “genetics,” with associated terms “population,” “resistance,” “qtl,” and “marker.” The blue cluster (70 terms) at the top of Figure 5 shows terms related to species and physiology/metabolism. In this last cluster, the model plant *Arabidopsis thaliana* is strongly associated with metabolism. However, being a model species, *A. thaliana* is also linked with both red (“growth” term) and green (“genes” term) cluster, remarkably bridging these two clusters. Other plant species like rice and *Triticum* are mainly associated to the green cluster reflecting the fact that these plants are highly studied for “plant breeding” and genomic selection.

**Figure 6:**
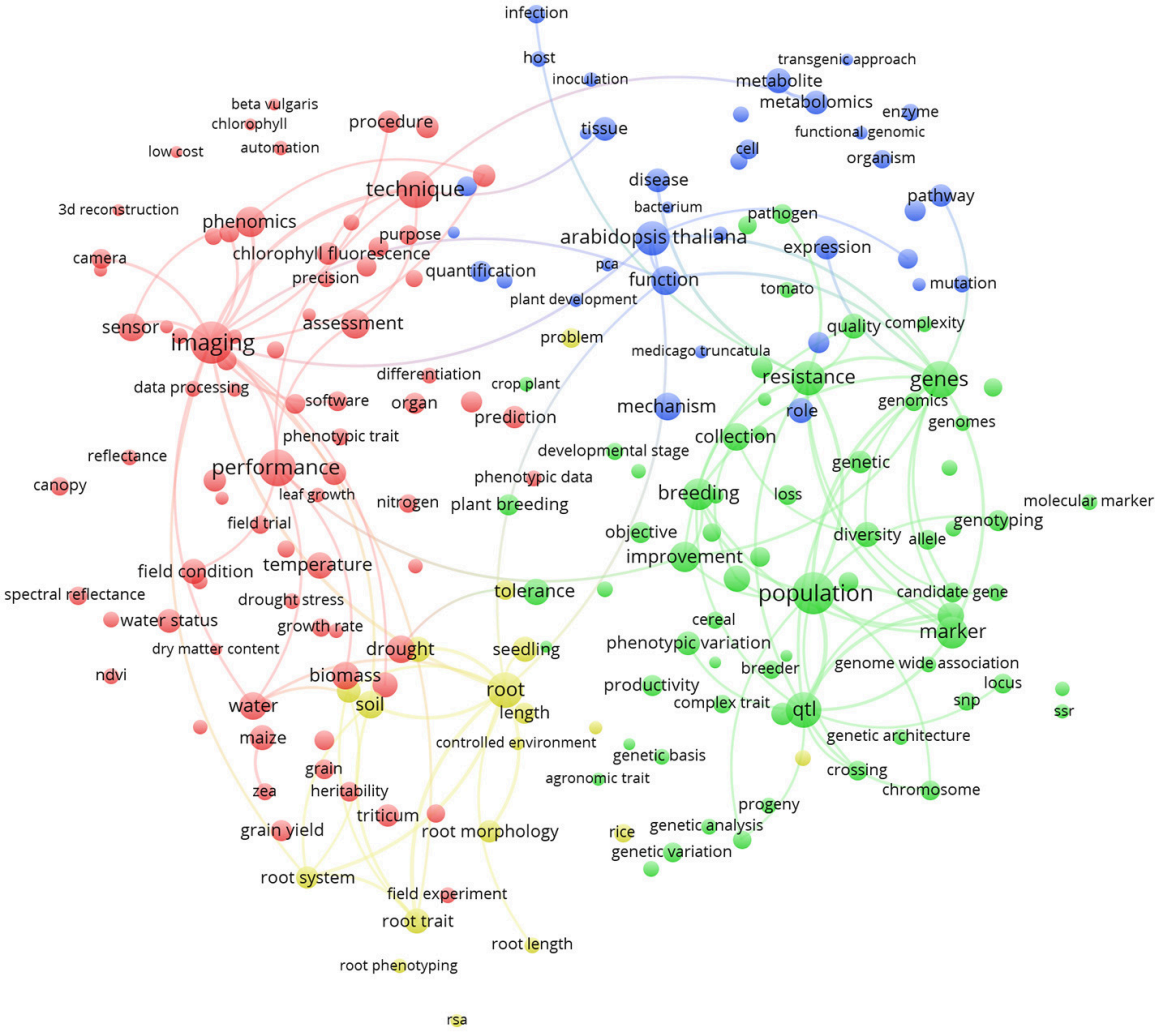
Term clustering map based on the Scopus plant phenotyping publications from the 5 most publishing EU countries (Germany, France, United Kingdom, Italy, Spain). Different colors (red, green, blue, and yellow) represent the terms belonging to different clusters. The size of the term is based on the number of occurrences. The connecting lines indicate the 100 strongest co-occurrence links between terms.

Similarly, Figure 6 represents the term map based on plant phenotyping publications from the 5 most publishing EU countries (Germany, France, United Kingdom, Italy, Spain; 755 papers). It could be noticed that, being a sub-group of the term map in Figure 5, green and red clusters are very similar (at least considering the general meaning). We observe that *Arabidopsis thaliana* (blue cluster; indicating plant physiology) is the most represented plant species (8.5% of the world total plant phenotyping papers) that is under investigation (relative larger circle in EU; 10.7% of the EU plant phenotyping papers). This points toward the observation that in the EU, research studies are mainly based on a model plant based laboratory context. Root phenotyping is represented in an additional cluster (yellow). Imaging (red cluster) is very important in the EU and cross linked with many other terms. Characterization of root architecture in soil-based assays in the lab and in the field remains challenging, and any useful methodology should also be exploited in specific chain combinations. Methodologies to study root growth and architecture in 2D and 3D are a frontier in plant phenotyping (Fiorani and Schurr, 2013).

**Figure 7:**
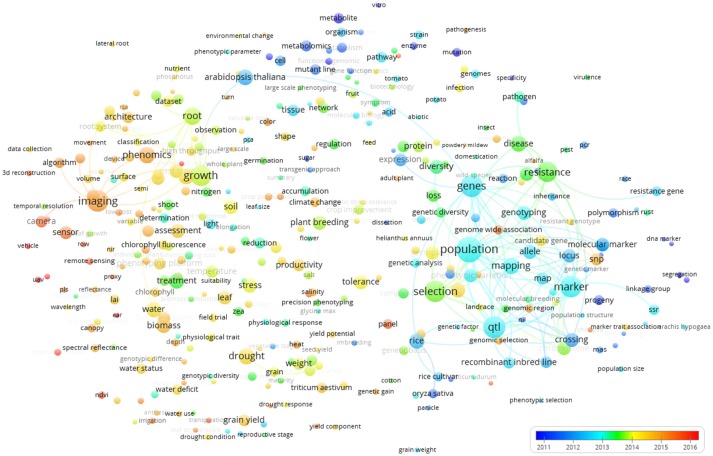
Term map indexed *per* publication year based on all the Scopus plant phenotyping publications. The time scale is represented by different colors. The size of the term is based on the number of occurrences. The connecting lines indicate the 100 strongest co-occurrence links between terms.

Figure 7 shows the world *term year map*. The colors show that terms used in early publications are mainly those represented by the blue and green clusters in Figure 5. These are terms related to genetics and metabolism that represent more “traditional” applications of plant phenotyping. The terms covered in more recent publications were rather related to the words imaging and plant/ environment interaction, as also identified by the red cluster of Figure 5. This clearly indicates a technology-driven change that takes advantage of progresses in ICT technologies and data analysis. The time stamp of the terms imaging, camera, phenomics, and sensor illustrate that high throughput plant phenotyping has a huge technological bias and a set of key technologies at hard and software level was required for the takeoff of plant phenotyping (image analysis and data storage management, automation, and robotics). Once they were developed, the publications started popping up. This technological shift is interestingly associated to a shift of the studied species from *Arabidopsis thaliana*, rice, potato, and tomato to *Zea mais, Triticum*, barley, and chickpea (Figure 7).

**Figure 8:**
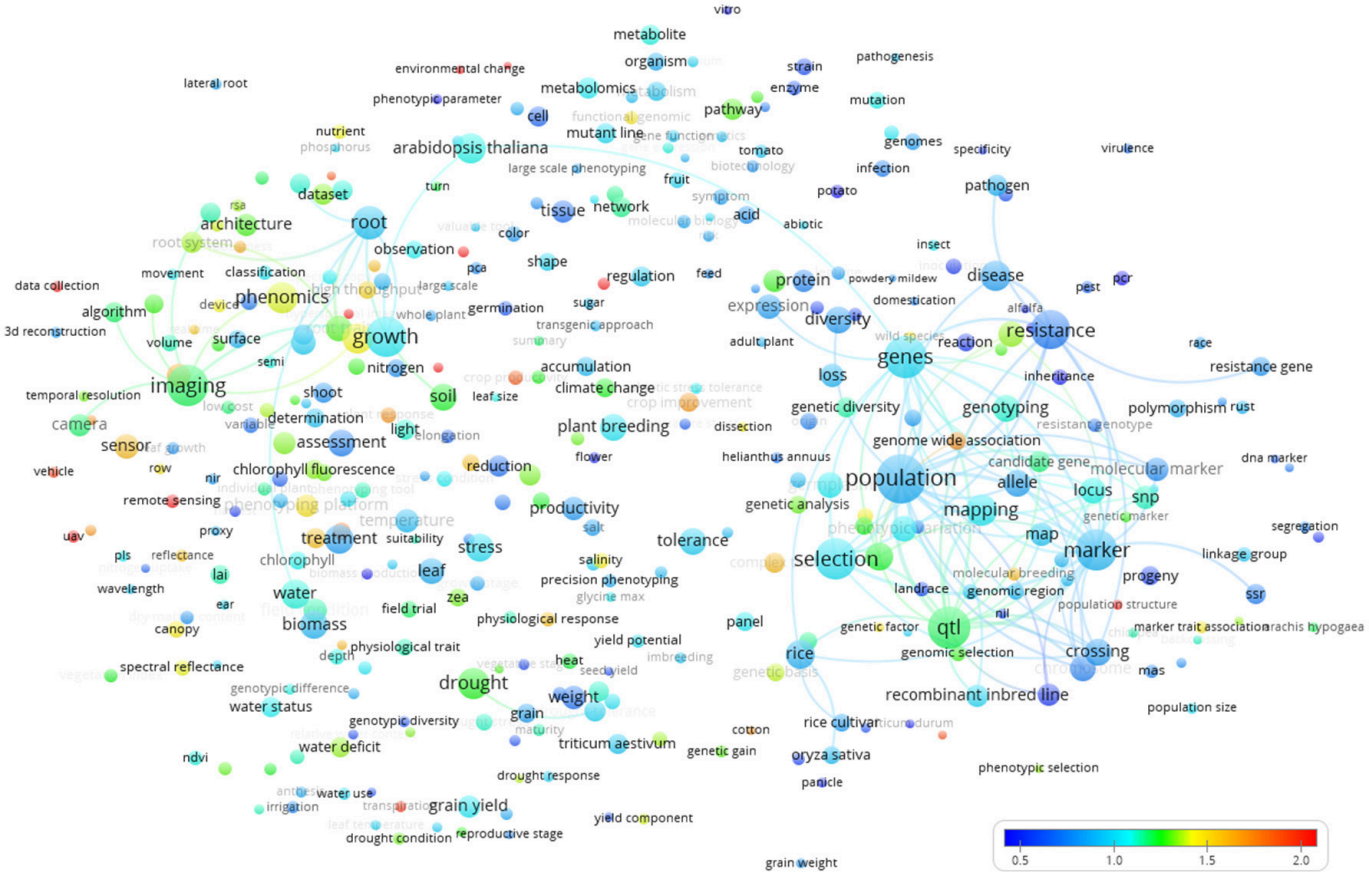
Term map based indexed per citation rate based on all the Scopus plant phenotyping publications/citations. The normalized citation rate scale is represented by the different colors. The size of the term is based on the number of occurrences. The connecting lines indicate the 100 strongest co-occurrence links between terms.

The world *term citation map* (Figure 8) revealed “food security,” “precision agriculture,” “plant physiology,” “cotton,” “UAV” (Unmanned Aerial Vehicle), “complex trait,” “canopy,” and “sensor” as highly cited terms (red colors).

**Figure 9:**
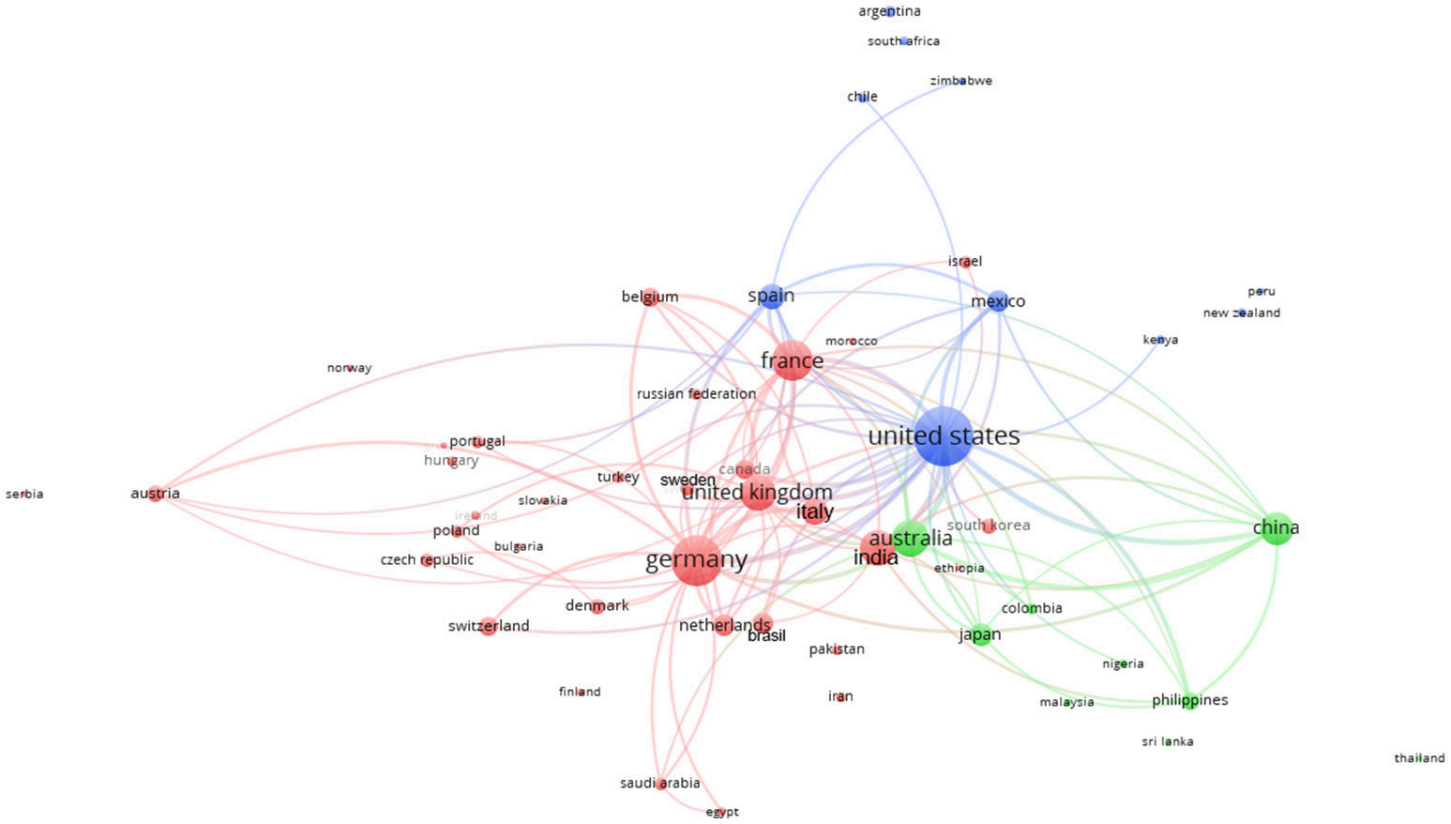
Collaborative clustering map of the countries based on the affiliations of co-authorships in Scopus plant phenotyping publications. Different colors represent different collaboration clusters. The size of the country is based on the number of publications. The connecting lines indicate the 100 strongest co-occurrence links between countries.

Figure 9 represents the collaborative clustering of the countries. It is possible to observe how the 3 clusters are polarized by the EU, USA, and Australia. The clusters represent the main collaborations (same color): EU (red cluster) with all the EU countries (except Spain) together with Turkey, Russia, Canada, Norway, Serbia, India, Brazil and other countries; USA (blue cluster) with Mexico, Spain, and other countries; Australia (green cluster) with China, Japan, and other countries. It is also possible to observe some strict collaborations among the clusters (many linkages).

**Figure 10:**
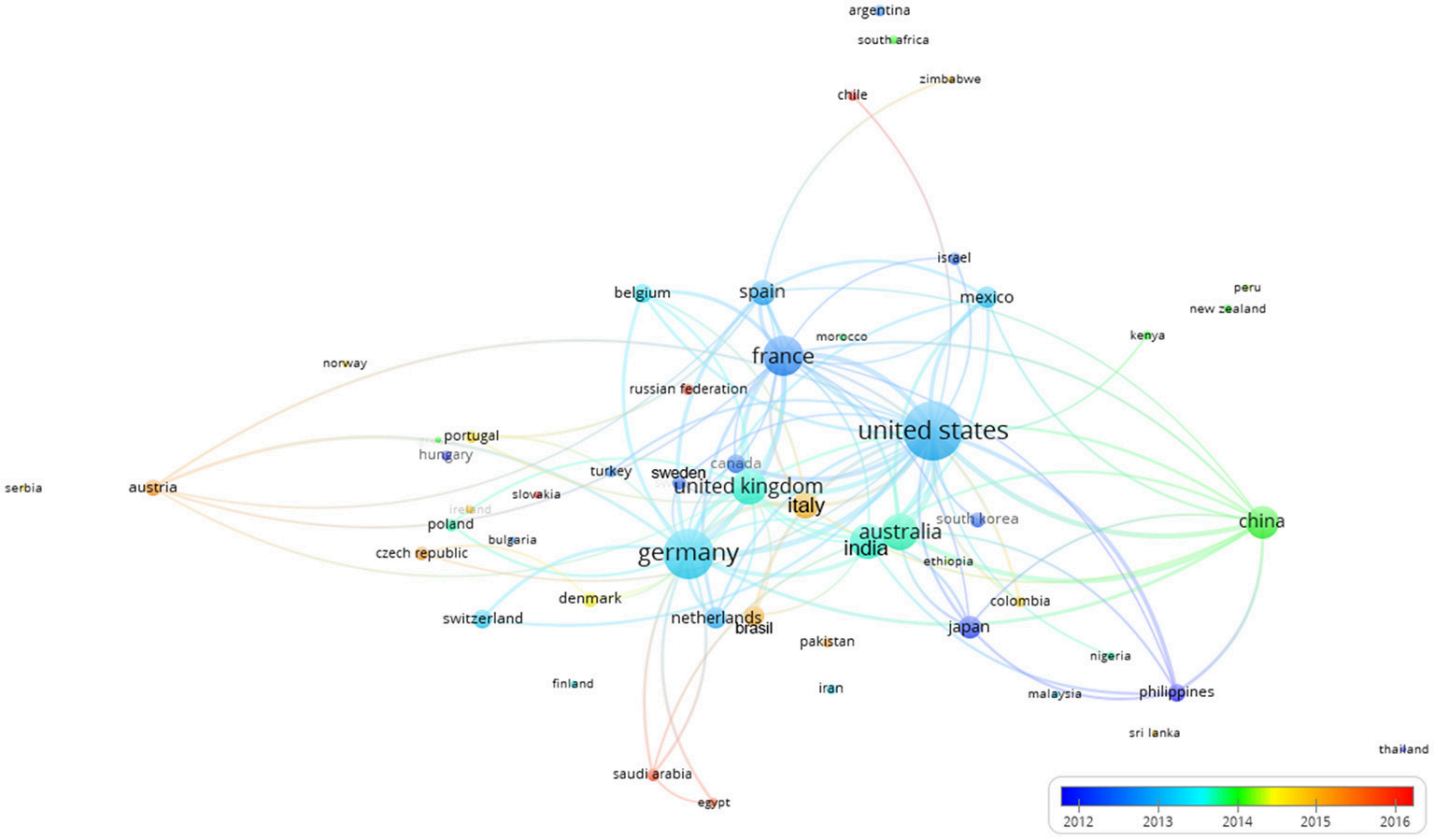
Collaborative clustering map of the countries indexed by time. The scale represented the earlier (blue) or more recent (red) mean year considering the period when the authors of each country published their papers. The connecting lines indicate the 100 strongest co-occurrence links between countries.

Figure 10 shows the collaborative clustering map of the countries indexed by time. The USA, France and Germany are the most represented in terms of number of publications (size of the indicator) and those collaborations were established rather early. China, Australia, India, and the UK (green colors) join in later with respect to pioneer countries such as (Germany, France and USA). In a more recent phase (reddish) also Italy and Brazil appear in the literature as collaborators.

Is there a relation between the proportion of the country that is dedicated to agriculture (FAO statistics) and the intensity of phenotyping research? Yes, countries more involved in plant phenotyping research development tend to have a low ratio (always lower than 7%). For example, EU and USA (leaders of plant phenotyping research papers) score both a value of 4.8% for this ratio. The first country, in terms of plant phenotyping publications, having a ratio higher than 7% is Saudi Arabia (8.1%; 21 plant phenotyping publications). However, this also indicates the sensitivity of the analysis at low numbers if highly cited researchers move countries.

## Conclusion and Future Perspectives

Modern plant phenotyping relies on a couple of rapidly developing pillars: (i) non-destructive measurements to be able to follow a trait over time; (ii) high-throughput measurements, to be able to screen at similar conditions many genotypes. Initially, considerable progress has been made in molecular and genetic analysis of model plants (*Arabidopsis thaliana*) and model crops (rice, tomato, etc.) for genetic models. Phenotyping, as an emerging science, will provide information on how genetics, epigenetics, environmental pressures, and crop management (farming), shape the phenotype of plants and guide selection toward productive plants that are suitable for their environment. But it needs to develop further, and to do so, there is a strong need for dedicated infrastructures providing tools and resources for phenotyping the valuable genomic resources available. This requires a multidisciplinary team of scientists with expertise in (i) sensor development, automation and usage, (ii) –omics in the broadest sense, (iii) plant ecology, physiology, pathology, and interactions with other organisms and (iv) (bio)informatics and statistics. As shown, the knowledge is currently still concentrated in key countries that have the infrastructures. Luckily several initiatives arouse to integrate and disseminate the knowledge and share the infrastructures. For example, the COST Action FA1306 (http://www.cost.eu/COST_Actions/fa/FA1306), the projects EPPN (http://www.plant-phenotyping-network.eu/) and EPPN2020 (https://eppn2020.plant-phenotyping.eu/), and the ESFRI-project EMPHASIS (European Infrastructure for multi-scale Plant Phenomics and Simulation for food security in a changing climate; https://emphasis.plant-phenotyping.eu/), jointly aim to establish pan-European excellence in plant phenotyping by synergistically develop relevant technologies, critical mass, infrastructures, and access. On a global scale an increasing number of national initiatives align phenotyping efforts in USA and Canada (NAPPN http://nappn.plant-phenotyping.org/), China (www.APPP-con.org), and others. Globally, the International Plant Phenotyping Network (IPPN; https://www.plant-phenotyping.org/) develops integrated approaches beyond the national and regional perspectives. Plant phenotyping should become a high priority in many countries. We show that phenotyping - a challenging multidisciplinary science - is taking off and has made the leap from research on the model plant Arabidopsis to real crops. This analysis shows that it is possible to grow, to share and to collaborate. As the first bibliometric study of plant phenotyping research, this analysis is exploratory, but sets the status of the research landscape. Despite limitations (bibliometric mapping constrains) the term map analysis represents a valid tool to support experts (countries) to improve their knowledge and orient them on novel fields. Future studies should address related issues such as the analysis of collaborations among different actors (*i.e*., authors, organizations and countries) as well as a deeper characterization of the research carried out in different geographic regions.

## Author Contributions

CC performed the database search. CC and PM performed the statistical analyses. CC and SC wrote the manuscript. All authors contributed to the discussion and approved the final manuscript.

### Conflict of Interest Statement

The authors declare that the research was conducted in the absence of any commercial or financial relationships that could be construed as a potential conflict of interest.
